# T lymphoblastic lymphoma with BCR-ABL negative chronic myeloid leukaemia: a novel association

**DOI:** 10.3332/ecancer.2021.1221

**Published:** 2021-04-22

**Authors:** Mahwish Faizan, Saadia Anwar, Rahat Ul Ain, Huma Zafar, Nazish Saqlain, Zunaira Rathore

**Affiliations:** 1 Department of Paediatric Haematology/Oncology, The Children’s Hospital & Institute of Child Health, Ferozepur Road, Lahore 54400, Pakistan; 2 Department of Haematology, The Children’s Hospital & Institute of Child Health, Ferozepur Road, Lahore 54400, Pakistan; 3 Department of Histopathology, The Children’s Hospital & Institute of Child Health, Ferozepur Road, Lahore 54400, Pakistan

**Keywords:** lymphoblastic lymphoma, children, atypical chronic myeloid leukaemia

## Abstract

Lymphoblastic lymphoma and chronic myeloid leukaemia (CML) are two distinct neoplasms with different pathogenesis and clinical presentation. We hereby share a challenging case of a child presenting with fever, leucocytosis, generalised lymphadenopathy and massive splenomegaly. He was diagnosed as having novel association of concurrent T-lymphoblastic lymphoma diagnosed on cervical lymph node biopsy with BCR-ABL negative CML on bone marrow aspirate. The study of more such cases is needed for optimal patient management.

## Introduction

Acute lymphoblastic leukaemia/lymphoma (LBL) is a clonal hematopoietic stem cell disorder of B or T cell origin. LBL comprise approximately 30% of childhood non Hodgkin lymphomas. Chronic myeloid leukaemia (CML) constitutes 2%–3% of leukaemia in children. Atypical CML (BCR-ABL negative) is a challenging myeloid malignancy with features of both myeloproliferative and myelodysplastic syndromes. The diagnosis of both myeloid and lymphoid neoplasms in a single patient, whether simultaneous or sequential, is extremely rare, with an overall incidence of less than 1% [1]. Our reported case has concurrent T lymphoblastic lymphoma and BCR-ABL negative CML, which have never been reported in the literature before.

## Case report

**Figure 1.**
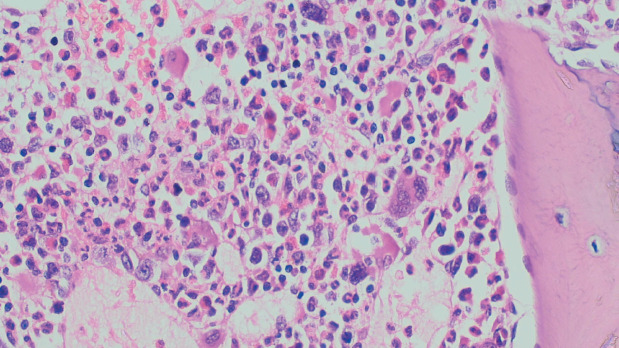
Bone marrow trephine biopsy.

A 9-year-old child presented to the oncology clinic in September 2020 with a 6 month history of fever, fatigue and gradually increasing bilateral neck swellings. There were no complaints of cough, respiratory distress, bone pains, petechiae or bruises. He was a pale looking, active and alert child having weight of 22 kg. A Bacillus Calmatte-Guerin (BCG) vaccine scar was present. He had generalised lymphadenopathy, largest were bilateral cervical lymph nodes 5 by 5 cm, discrete, firm, non-tender with normal overlying skin. His liver was enlarged 4 cm below right costal margin and spleen was enlarged 15 cm below left costal margin. The rest of systemic examination was unremarkable. His haemoglobin was 6.9 gm/dL, White blood cells (WBC) 168.4 × 10^3^/L, neutrophils 36%, lymphocytes 02%, eosinophil’s 07%, monocytes 02%, promyelocytes 02%, myelocytes 26%, metamyelocytes 16%, basophils 07%, blasts 02% and mean corpuscular volume (MCV) 80.1 fL, mean corpuscular hemoglobin (MCH) 26.4 pg, Peripheral blood film shows macrocytosis, anisocytosis and poikilocytosis. Nucleated red blood cells 03/100 WBC. No hemoparasites were identified. Platelets 72,000/L. serum uric acid 7.2 md/dL, potassium 4 mmol/L, latcate dehydrogenase (LDH) 780. Echocardiography, hepatitis B & C screening, calcium, phosphate, liver and renal function tests were normal. CT neck, chest, abdomen showed bilateral cervical (largest measures 6 cm by 5 cm) , axillary, supraclavicular, mediastinal and abdominal lymphadenopathy, hepatosplenomegaly (L 15 cm, S 17.2 cm) and splenic parenchymal defects suggesting lymphoproliferative disorder with incidental note of left pelvi ureteral junction (PUJ) obstruction. Flow cytometry on bone marrow aspirate was inconclusive showing less than 5% blasts. Bone marrow aspirate showed hypercellular fragments showing active trilineage haematopoiesis. Plasma cells constitute 3% of nucleated marrow cells. No hemoparasites identified. Blasts cell constitute 05% of nucleated marrow cells. BM differential was myelocytes 40%, metamyelocytes 02%, erythroids 05%, neutrophils 45% and blasts cells 08%. Bone marrow trephine biopsy was adequate length with normal bony trabeculae, reduced fat spaces and 95% cellularity (Figure 1). Erythropoiesis was suppressed with hyperplastic myelopoiesis and megakaryopoiesis seen. Peripheral blood film and bone marrow findings were suggestive of chronic myeloproliferative neoplasm (MPN) most likely chronic myelogenous leukaemia in chronic Phase.

**Figure 2.**
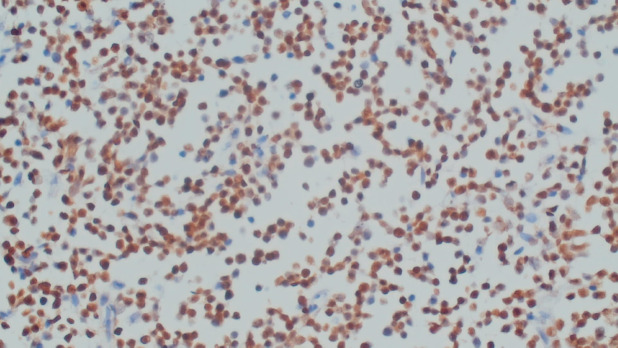
Cervical lymph node biopsy (terminal deoxynucleotidyl transferase/Tdt positive).

Excisional biopsy of cervical lymph node was pan T (CD3) positive, pan B (CD20) negative, Tdt positive, CD117 negative, cyclin D-1 negative and KI 67 High (>80%) consistent with T cell lymphoblastic lymphoma/leukaemia (Figure 2). BCR-ABL translocation was not detected on fluorescence *in situ* hybridization (FISH) and real-time polymerase chain reaction (RT-PCR). Differential diagnosis considered included CML with Lymphoma, lymphadenopathy as an extramedullary manifestation of CML or myeloid sarcoma. Considering the rarity of association of T lymphoblastic lymphoma/leukaemia with BCR-ABL negative CML in paediatric age group, both the cervical lymph node biopsy and bone marrow aspiration biopsy were sent for external review, after discussion in tumour board meeting. Both reports corresponded to and confirmed the primary diagnosis of T lymphoblastic lymphoma with BCR-ABL negative CML which is unheard of in the paediatric population. The family was appropriately counselled. The child was started on tumour lysis prophylaxis, Hydration and pneumocystis jerovecci prophylaxis. The literature was searched to make optimal treatment plan for this child. There was a paucity of data to make exact recommendation of the treatment. The family was counselled about bone marrow transplant when his lymphoblastic lymphoma was in remission, to which they did not consent. The child was started on UKALL 2011 Regimen C protocol. Four Drug Induction completed on 1st January 2021. Currently, the child is on Capizzi Interim Maintenance chemotherapy and doing well. Post induction workup showed haemoglobin was 10.8 gm/dL, WBC 24.7 8*10^3^/L, neutrophils 50%, lymphocytes 06%, eosinophil’s 04%, monocytes 05%, promyelocytes 02%, myelocytes 24%, metamyelocytes 9%, basophils 01% and blasts 01%. CT scan neck chest and abdomen show few subcentimeter size lymph nodes in neck, more on left side with subcentimetric axillary and supraclavicular, mesenteric lymph nodes. Right kidney is normal. Left kidney shows PUJ obstruction. Liver enlarged 14 cm and spleen measures 14.2 cm markedly enlarged. Repeat bone marrow (induction day 8 & day 29) still favour MPN likely CML in chronic phase. Currently, clinically, he is doing well with no palpable lymphadenopathy; however, splenomegaly persists 4 cm (from 15 cm baseline) below left costal margin, though markedly regressed. His lymphoblastic Lymphoma seems to be in remission while atypical chronic myeloid leukaemia (aCML) in chronic phase persists.

## Discussion

Lymphoma is the third most common childhood malignancy accounting for 10%–12% of childhood cancers. Non-Hodgkin lymphoma (NHL) account for 7% of cancers in children <20 years of age [2]. T-cell lymphoblastic lymphoma/leukaemia (T-LBL) is the most common paediatric T cell Lymphoma. They develop in lymphoid tissues such as lymph nodes and spleen or outside of lymphoid tissues like gastrointestinal tract, liver, nasal cavity, skin etc. CML is rare in children and constitutes 2% of all leukaemias in children younger than 15 years and 9% of all leukaemias in adolescents between 15 and 19 years [3]. About 90%–95% of children with CML have a genetic alteration called the Philadelphia chromosome. aCML is a rare myelodysplastic syndrome/MPN in children.

Morphology-based criteria are currently used to diagnose it. The main feature of aCML is the presence of neutrophilic leucocytosis and marked dysgranulopoiesis. Further diagnostic criteria include WBC ≥ 13 × 10^9^/L with ≥ 10% of immature granulocytes, and ≤ 20% blasts in the blood and the bone marrow [4, 5]. To rule out other MPNs, BCR-ABL1, platelet-derived growth factor receptor alpha (PDGFRA), platelet-derived growth factor receptor beta (PDGFRB), and fibroblast growth factor receptor (FGFR) rearrangements should be excluded in all cases [6].

There has been a case report of secondary malignancies in CML [7]. Fu *et al* [8] reported simultaneous diagnosis of T-LBL and BCR-ABL positive CML in two adult patients who were treated with imatinib and HYPER-CVAD chemotherapy. Simultaneous acute myeloid leukaemia (AML) and lymphoma has been reported in the literature and is more frequent than simultaneous CML and lymphoma [9]. Melikian *et al* [10] reported 20 adult patients with synchronous and metachronous tumors. There are also case reports of coexistence of T cell Lymphoblastic lymphoma co existing with atypical myeloproliferative disorder associated with t(8:13) (p21;q14) [11].

Our index patient fulfilled the criteria of both precursor T-NHL based on immunohistochemistry and typical morphology on lymph node biopsy and BCR-ABL negative CML. This association has not been previously described in the literature either in adults or children. Due to the rarity of this co existence, little is known about pathogenesis and optimal treatment plan.

Precursor T-NHL is an aggressive malignancy but the development of intensified T-ALL–focused protocols has resulted in significant improvements in outcome in children [12]. No standard of care is currently available for the management of co existent aCML. Drugs called tyrosine kinase inhibitors are the first line of treatment for BCR-ABL positive CML. However, the only curative treatment for atypical CML remains haematopoietic stem cell transplant (HSCT) [13]. Participation in clinical trials should be considered in all cases. Different treatment options like hypomethylating agents (azacytidine or decitabine) [14], Hydroxy urea, Interferon alpha, AML like therapy or targeted therapies (Janus kinase gene (JAK) inhibitor ruxolitinib, the SRC (proto-oncogene tyrosine-protein) kinase inhibitor dasatinib, and the MEK inhibitor trametinib) have been used with variable results in patients not suitable for bone marrow transplant [15, 16]. The prognosis of the concurrent bi-lineage malignancies is generally poorly understood and chemotherapy is necessary. More research is required to optimize management for such children.

## Conclusion

T lymphoblastic lymphoma with atypical CML is an unusual association. The T LBL component regressed completely in the index child after treatment with T ALL based therapy. More data is required to optimize management for such children.

## Conflicts of interest

No conflicts of interest.

## Funding

No funding was received for this case report for any purpose.
